# Impact of Cherry Powder as a Natural Antioxidant on Quality, Oxidative Stability and Microbial Activity of Ready-to-Eat Beef Patties

**DOI:** 10.3390/foods15091483

**Published:** 2026-04-24

**Authors:** Fatimah Munishmehdi Umatiya, Zubayed Ahamed, Naomi Vinden, Chawalit Kocharunchitt, Roger Stanley, Md Saifullah

**Affiliations:** Centre for Food Safety and Innovation, Tasmanian Institute of Agriculture, University of Tasmania, Launceston, TAS 7250, Australia; fatimahu@utas.edu.au (F.M.U.); zubayed.ahamed@utas.edu.au (Z.A.); naomi.vinden@utas.edu.au (N.V.); chawalit.kocharunchitt@utas.edu.au (C.K.); roger.stanley@utas.edu.au (R.S.)

**Keywords:** natural antioxidant, cherry powder, oxidative stability, ready-to-eat meat products, colour, microbial activity

## Abstract

Oxidation and related quality deterioration remain a significant challenge for the food industry. Antioxidants are widely used to address these issues, and natural antioxidants are explored as alternatives to synthetic counterparts due to health concerns. This study investigated the impact of cherry powder (CP) on the oxidative stability and quality of ready-to-eat meat products. Beef patties were made and processed by sous vide cooking, then stored at 22 °C to simulate shelf-stable conditions, divided into six treatments: a negative and a positive control, and four CP concentrations (1%, 3%, 5%, and 7%). The antioxidant activities (TPC, FRAP, and DPPH), lipid oxidation, and microbial activity were measured over a 7-day storage period, along with the impact of processing on these parameters. CP significantly (*p* < 0.05) enhanced oxidative stability, reducing lipid oxidation compared to controls. Antioxidant activity was significantly (*p* < 0.05) affected by processing and decreased post-processing and storage, except for DPPH radical scavenging, which remained stable. CP showed no significant antimicrobial effect, as microbial counts in all treatments exceeded 10^4^ cfu/g by day 1, indicating elevated microbial levels and a decline in product quality, although they remained below the level generally considered unsatisfactory for ready-to-eat meat products. Colour analysis showed significant (*p* < 0.05) variations in L*, a*, and b* values post-processing and during storage. Overall, the CP addition improves the colour and oxidative stability and could be a potential source of antioxidants to maintain the quality of meat products.

## 1. Introduction

Meat and meat products are known to be excellent natural sources of essential proteins, amino acids, minerals such as iron and zinc, vitamins, especially vitamin B12, and other nutrients [1]. These nutrients are vital to a healthy diet, making meat an essential part of the global food chain. There has been a gradual increase in global meat consumption over the past 20 years, rising from 58 million to 360 million tons annually. One crucial aspect of the meat industry’s ready-to-eat (RTE) meat processing is that it provides consumers with a convenient and safe option for meat products.

The growing demand and financial benefits of meat products means that meat spoilage remains a key concern for the industry. Lipid oxidation, digestive enzymes, and microbiological activity are the key factors involved in meat spoilage [2]. Oxidative deterioration is the second most common cause of meat spoilage, following microbial spoilage. Meat oxidation is a multi-step complex process. This involves several interdependent processes that depend on the composition of the meat, exposure to oxygen, light, and storage temperatures. It leads to chemical reactions that result in undesirable changes, such as discolouration of meat and changes in texture and appearance [3,4]. In particular, lipid oxidation is a significant issue because it generates volatile compounds that cause rancidity and off flavours, thereby affecting the shelf-life and sensory appeal of meat products [5]. Furthermore, the toxic compounds formed during oxidation are known to have mutagenic, cytotoxic, and oxidative effects on the human body [6,7,8].

The negative effect of oxidation highlights the importance of having an effective technique to prevent oxidative spoilage while maintaining the quality and safety of meat products. The main technique used to mitigate oxidation is the addition of antioxidants to meat and meat products [3,5,9,10,11]. The mechanisms by which antioxidants prevent oxidation are mainly based on breaking chain reactions, scavenging free radicals, chelating metal ions that catalyse oxidation, and reducing localised oxygen concentrations, thereby inhibiting lipid and protein oxidation in the meat matrix [11]. Antioxidants are compounds that prevent oxidation reactions and are broadly categorised as synthetic or natural. Commonly used synthetic antioxidants include butylated hydroxytoluene (BHT), butylated hydroxyanisole (BHA), propyl gallate (PG), tertiary butylhydroquinone (TBHQ), and synthetic tocopherol (vitamin E), as well as nitrites, nitrates and phosphates [2,12]. However, extended and long-term use of synthetic antioxidants may have possible health concerns such as allergic reactions, pregnancy complications, disorders in children, and potential carcinogenic effects [8]. Thus, there is a growing need to explore suitable natural alternatives to synthetic antioxidants. Additionally, although the allowable level of synthetic antioxidants is as low as 0.02% of fat in food, consumers are increasingly seeking products with organic and natural antioxidants [13].

Many natural antioxidants are products of plant origin that contain high concentrations of polyphenolic compounds, making them an excellent alternative to synthetic antioxidants [8]. The antioxidant potential of these natural antioxidants sourced in the form of extracts or powder or pulp from different sources such as fruits (grape, plum, cherry, pomegranate), vegetables (mint, potato, curry, etc.) and herbs and spices (clove, cinnamon, mint, thyme, oregano, rosemary, sage, etc.) are investigated and reported by several studies [14,15,16,17,18,19].

One of the fruits known to be rich in bioactive compounds with high antioxidant properties is the sweet cherry (*Prunus avium*). Phenolic compounds, flavonoids, and anthocyanins present in cherry powder can effectively scavenge free radicals and inhibit oxidative processes in various food matrices [20]. These phenolic compounds extend shelf-life and maintain meat quality primarily through radical scavenging, metal ion chelation, and inhibition of lipid and protein oxidation, thereby preserving colour, texture, and flavour. Furthermore, cherries are widely available, consumer-acceptable, and have been reported to maintain colour and oxidative stability in food matrices, making them particularly suitable for RTE meat products. In addition to its high nutrient content, cherry also exhibits numerous bioactivities, including anti-inflammatory, anti-tumour, anti-cancer, and antidiabetic [20,21]. Thus, it is believed that using cherry as a natural antioxidant in meat will not only enhance oxidative stability and maintain product quality but also meet consumer preferences.

There is extensive literature that focuses on the ability of cherries and their antioxidant capacity to inhibit oxidation in raw meat products. However, the existing research lacks comprehensive studies on the application and stability of cherry-derived antioxidants for ready-to-eat meat processing, particularly in the context of sous vide treatment. There is a noticeable gap in understanding the stability of cherry antioxidants within beef patties subjected to these specific processing techniques. Additionally, there is a lack of data regarding the shelf-life stability of such antioxidants in ready-to-eat meat products. This critical information is essential for determining the long-term efficacy of cherry-derived antioxidants as natural preservatives in the challenging environment of processed meat products, thus ensuring their safety, quality, and consumer acceptance over extended periods of storage and consumption. Addressing these specific knowledge gaps will significantly contribute to the development of effective and sustainable preservation strategies for ready-to-eat meat products.

## 2. Materials and Methods

### 2.1. Chemicals and Sample Materials

Freeze-dried cherry powder (CP) was supplied by Forager Foods Co. (Launceston, TAS, Australia). Upon receipt, CP was stored in vacuum packaging at 4 °C in the dark to prevent degradation of bioactive compounds. Fresh beef mince was from a local supermarket near the University of Tasmania (UTAS). The lean-to-fat ratio was approximately 80% to 20%.

Ferric chloride (FeCl_3_), 2,4,6-Tri(2-pyridyl)-s-triazine (TPTZ), 2,2-diphenyl-1-picrylhydrazyl (DPPH), (±)-6-hydroxy-2,5,7,8-tetramethylchroman-2-carboxylic acid (trolox), trichloroacetic acid (TCA), Folin–Ciocalteu’s reagent, thiobarbaturic acid (TBA), gallic acid, sodium carbonate, and methanol of analytical grade were purchased from Merck Life Science Pty Ltd. (Bayswater, VIC, Australia).

### 2.2. Beef Patty Preparation

Lean meat 80% and 20% fat was minced in a meat grinder and used to make patties. A 100 g sample of minced beef was used to make the patty using a patty maker (Hamburger Machine BT10, BERKEL OMAS, Varese, Italy) to ensure uniform shape and thickness in the Micro Research lab at the Tasmanian Institute of Agriculture, UTAS. Six different groups of patties were made. One group with no added antioxidants was used as the negative control (NC), PC as the positive control with 0.01% BHT added, 1% CP for 1% *w*/*w* CP, 3% CP for 3% *w*/*w* CP, 5% CP for 5% *w*/*w* CP, and 7% CP for *w*/*w* 7% CP. A total of 96 patties were prepared.

### 2.3. Cooking Method

After formation, the patties were placed into transparent, food-grade, sous vide-compatible vacuum bags (150 × 200 × 0.08 mm; Tas Catering Supplies, Hobart, TAS, Australia) and vacuum-sealed using a commercial vacuum sealer. The vacuum-packaged patties were cooked in a temperature-controlled shaking water bath (PLS20, Ratek Instruments Pty Ltd., Boronia, VIC, Australia) at 70 ± 1 °C for 10 min. The water bath temperature was continuously monitored to ensure consistency throughout the cooking process. Following cooking, the vacuum-packaged patties were removed from the water bath, surface moisture was gently wiped off, and samples were immediately transferred to an incubator set at 22 ± 1 °C in the dark. This condition was used to simulate accelerated storage at room temperature rather than typical commercial storage. Samples were collected and analysed on days 0, 1, 4, and 7. For day 0 measurements were conducted after allowing the samples to equilibrate for 1 h post-cooking.

### 2.4. Determination of Lipid Oxidation

The 2-thiobarbituric acid-reactive substances (TBARS) assay was employed to assess lipid oxidation levels in beef patties, with slight modifications [22]. Extraction solution for samples was prepared fresh by mixing 10 mL of 7.5% (*w*/*v*) TCA solution with 2 mL of 1% BHT in ethanol (*w*/*v*). Two grams of the meat sample were combined with 10 mL of extraction solution in a sterile 170 × 300 mm blender bag (Westlab, Mitchell Park, VIC, Australia) and homogenised using a stomacher (Colworth 400, Colworth Science Park, London, UK) for 2 min. The homogenate was then transferred to a test tube, centrifuged at 6000 rpm for 10 min using an Eppendorf AG 5811 centrifuge (Eppendorf, Hamburg, Germany), and filtered through 90 mm qualitative filter paper (Westlab, Mitchell Park, VIC, Australia). Next, 1 mL of the supernatant was mixed with an equal volume of 20 mM thiobarbituric acid (TBA) solution in test tubes. The test tubes were heated in a water bath at 90–95 °C for 60 min. Absorbance at 532 nm was measured using a UV-Vis microplate reader (SPECTROstar Nano, BMG Labtech Pty. Ltd., Mornington, VIC, Australia). A blank solution was prepared by mixing 1 mL of the sample solution with distilled water. The average of three absorbance readings were used to calculate TBARS values, expressed as malondialdehyde (MDA) mg/kg of meat, using a standard curve generated with 1,1,3,3-tetraethoxypropane (TEP).

### 2.5. Antioxidant Assays

#### 2.5.1. Sample Preparation

The sample extract for antioxidant analysis was prepared following the method with slight modifications [23]. Four grams of the sample were mixed with 20 mL of a 50:50 v/v methanol: deionised water solution in a 170 × 300 mm sterile blender bag (Westlab, Mitchell Park, VIC, Australia) and homogenised using a stomacher (Colworth 400) for 2 min. The homogenate was left to sit for 10 min before being centrifuged at 6000 rpm for 10 min using an Eppendorf AG 5811 centrifuge (Hamburg, Germany). The resulting supernatant was then filtered through 90 mm qualitative filter paper (Westlab, Mitchell Park, VIC, Australia) for analysis. All samples were stored at 4 °C during the analysis.

#### 2.5.2. Ferric Reducing Antioxidant Assay

The FRAP assay was performed following the methods with slight modifications [23,24]. The FRAP reagent was prepared by mixing 50 mL of 300 mM sodium acetate buffer with 5 mL of 20 mM ferric chloride solution and 5 mL of 10 mM TPTZ solution in a 10:1:1 ratio. The reagent was then incubated at 37 °C for 10 min before use. One hundred microliters of the sample solution were added to 1900 µL of the prepared FRAP reagent, and the mixture was incubated in the dark at 37 °C for 30 min. Absorbance was measured at 593 nm against a blank, which was prepared similarly by substituting 100 µL of the extraction solution for the sample solution. The results were expressed as µmol Trolox Equivalent per 100 g of sample.

#### 2.5.3. DPPH Radical Scavenging Assay

The free radical scavenging capacities of the antioxidants were determined using the DPPH [23]. Twenty microliters of the sample extract were mixed with 180 µL of DPPH working solution. The reaction mixture was allowed to stand in the dark at 37 °C for 60 min. Absorbance was measured at 517 nm in a 96-well plate using a microplate reader (SPECTROstar Nano, BMG Labtech Pty. Ltd., Mornington, VIC, Australia). The results were expressed as mg Trolox Equivalent (TE) per 100 g of sample.

#### 2.5.4. Total Phenolic Content (TPC) Determination

Total phenolic content was measured using this method with slight modifications [9]. Twenty microliters of sample extract were mixed with 100 µL of Folin–Ciocalteu (FC) reagent. Subsequently, 80 µL of 7.5% (*w*/*v*) sodium carbonate solution was added. The mixture was incubated in a water bath at 45 ± 1 °C for 30 min. After cooling in an ice water bath, the absorbance of the solution was measured at 765 nm in a 96-well plate, using a microplate reader (SPECTROstar Nano, BMG Labtech Pty. Ltd., Mornington, VIC, Australia). Gallic acid was used as a standard to prepare the calibration curve, and the results were expressed as gallic acid equivalents (GAE) in mg per g of sample.

### 2.6. Total Viable Count (TVC)

The total viable count (TVC) for anaerobic bacteria was determined using spread plate technique, for all samples [16]. Sample (2 g) was mixed with 18 mL of sterilised 0.1% peptone water in a sterile 170 × 300 mm blender bag using aseptic techniques. The mixture was homogenised in a stomacher for 2 min. Serial dilutions were performed using 0.1% peptone water, and 100 µL of the appropriate dilution were spread onto Tryptone Soy Agar (TSA, Oxoid) plates. Plates were incubated at 25 °C for 3 days under anaerobic conditions. Colony counts within the range of 30–100 CFU were recorded, and the TVC was expressed as log CFU/g.

### 2.7. Instrumental Colour and pH

The colour values of the samples were measured in terms of lightness (L*), redness (a*), and yellowness (b*) using a CR-400 Chroma Meter (Konica Minolta Sensing Americas, Inc., Ramsey, NJ, USA). Prior to measurement, the instrument was calibrated using a standard white plate provided by the manufacturer. Each sample was measured five times, and the average value was recorded. The pH of the samples was determined using a digital pH meter with a penetration probe. The probe was used to measure the pH of the sample mixture by directly immersing the electrodes. The pH meter was calibrated each sampling day before and between every five samples using standard buffer solutions of pH 4.0 and 7.0.

### 2.8. Statistical Analysis

Data analysis was performed using IBM SPSS Statistics (Version 29.0.2.0). A one-way ANOVA with Tukey’s post hoc test for multiple comparisons was used to evaluate the effects of treatment and storage time on oxidation levels in the samples. The antioxidant stability of the samples, as well as variations in pH and total viable count (TVC) over the storage period, were also analyzed using one-way ANOVA to assess changes across time points within each treatment. Paired t-tests were conducted to evaluate changes in colour parameters (L*, a*, and b* values) and antioxidant capacity before and after thermal processing. All statistical analyses were considered significant at a *p*-value ≤ 0.05.

## 3. Results and Discussion

### 3.1. Lipid Oxidation

**Figure 1:**
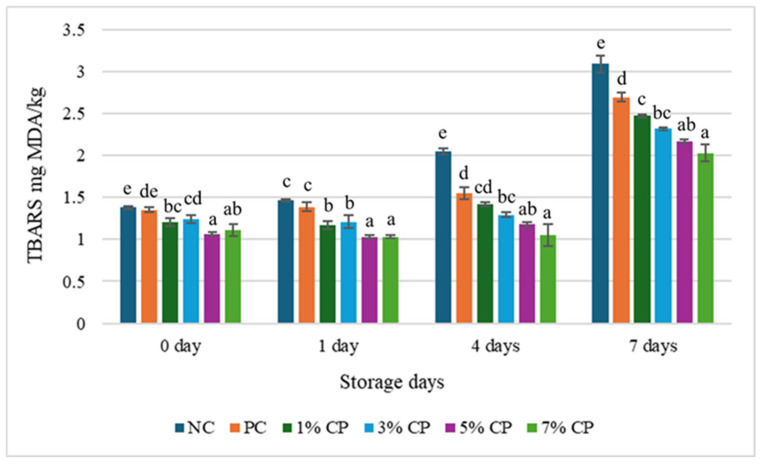
Changes in mean TBARS value among the treatments in cooked beef patties stored at 22 °C for 7 days. Error bars represent the Standard deviation. Different letters (a–e) for each day cluster represent significant differences (*p* < 0.05) among the treatments.

The effect of a natural antioxidant on lipid oxidation level, measured by TBARS value, in cooked beef patties stored at 22 °C for 0, 1, 4, and 7 days is depicted in Figure 1. Overall, TBARS values increased gradually until day 7, when they reached the highest values across all treatments, although there were fluctuations within each treatment on each day. On days 0 and 1, there was no significant difference in TBARS values between the positive and negative control groups (*p* > 0.05). The lowest MDA value was observed in samples on day 0 with 5% CP. Throughout storage, the two highest CP concentrations (5% and 7%) showed the lowest levels of oxidation, and there was no significant difference between these two treatment groups (*p* > 0.05). TBARS values on days 4 and 7 showed a sequence of NC > PC > 1% CP > 3% CP > 5% CP ≥ 7% CP.

The findings of our study for the initial storage days are similar to a study, who observed no significant differences (*p* < 0.05) on day 0 between the antioxidant-treated (cherry powder) and non-treated samples [25]. A possible reason for this might be the time required to activate the polyphenolic compounds and the interaction with pro-oxidants [26]. Over the 7-day storage period, for each treatment, the TBARS value increased significantly, with the highest value observed in the negative control (*p* < 0.05). The possible cause of the higher TBARS value in the negative control is the release of MDA, a secondary product of lipid oxidation [27]. Up to 1 day, no significant changes were observed for each treatment, except for 3% and 7% CP, where no significant changes were observed for up to 4 days (*p* < 0.05). All treatments with cherry powder exhibited lower MDA content than those with BHT, indicating that cherry powder was more effective at controlling oxidation levels than synthetic antioxidants like BHT at 0.01%. Our findings also align with those of a study, which reported that adding 3% and 5% cherry powder to Jiangsu-type sausage effectively inhibited lipid oxidation during refrigerated storage for 30 days [28].

### 3.2. Effect of Thermal Processing on Antioxidant Stability

Processing affects the bioactive compounds and antioxidant activity of the antioxidants [29]. The antioxidant stability of beef patties treated with CP was assessed using FRAP and DPPH assays, as well as TPC via the Folin–Ciocalteu method. Table 1 shows the effect of thermal processing on antioxidant stability and TPC stability. The ferric reducing activity of the CP was significantly affected by thermal processing (*p* < 0.05). Between treatments, the raw NC sample had the lowest FRAP value of 7.55 ± 0.60 TE µmol/g. In contrast, values for CP treatments ranged from 15.91 ± 0.79 TE µmol/g (1% CP) to 26.00 ± 0.28 TE µmol/g (7% CP), with 7% CP showing the highest reducing ability due to its high antioxidant content. After thermal processing the products, the FRAP value reduced to 11.61 ± 0.93 TE µmol/g from 24.41 ± 0.45 TE µmol/g. Except for the NC-treated group, all CP-treated groups showed significantly lower FRAP values (*p* < 0.05).

The DPPH radical-scavenging activity was substantially affected by thermal processing. A significant loss occurred in all the treatment groups after processing (*p* < 0.05). The uncooked NC group lost approximately 50% DPPH activity in processed samples. Furthermore, significant differences (*p* < 0.05) in DPPH values were observed among CP treatment samples due to varying CP concentrations. The highest DPPH value of 20.60 ± 0.25 TE µmol/g was observed in samples containing 7% CP, which decreased to 6.64 ± 0.03 TE µmol/g after processing, indicating a loss of almost 75% in DPPH activity. Although the 7% CP sample lost huge of its DPPH activity after thermal processing, the remaining antioxidant capacity (6.64 ± 0.03 TE µmol/g) is still substantial and likely sufficient to provide functional benefits. A study by on orange sweet potato and red rice found 16% and 23% decreases in DPPH values after boiling, respectively [29].

A similar trend was also observed for the TPC assay. TPC differed significantly (*p* < 0.05) with an overall decrease in values across all treatments post-processing. TPC values post-processing ranged from 2.04 ± 0.04 GAE mg/g (1% CP) to 3.55 ± 0.05 GAE mg/g (7% CP), which was significantly lower than raw samples, which ranged from 3.35 ± 0.05 GAE mg/g (1% CP) to 4.14 ± 0.07 GAE mg/g (7% CP). The decrease in antioxidant activity and TPC after processing is due to thermal processing, which leads to structural changes in phenolic compounds and a subsequent loss of antioxidant properties [30]. There was a greater reduction in DPPH activity (~68% in 7% CP) compared to FRAP (7%) and TPC (13%) after thermal processing of the equivalent sample. This discrepancy likely reflects the different mechanisms measured by each assay: DPPH primarily detects free radical scavenging, which can be more sensitive to structural changes in phenolic compounds during heating, whereas FRAP and TPC measure reducing power and total phenolics, which are partially retained. Another study also observed similar reductions in antioxidant activity due to heating [31], and there is a relationship present between lipid oxidation and antioxidant activity. Grassi et al. [24] reported a decrease in antioxidant activity of cooked beef compared to raw meat, due to structural changes in the meat’s muscle, leading to increased lipid oxidation. Previous studies also examined the effects of thermal treatment on antioxidant stability and concluded that heating led to a sequential reduction in phytochemical content and antioxidant activity [32,33].

### 3.3. Effect of Storage Time on Antioxidant Activity

**Figure 2:**
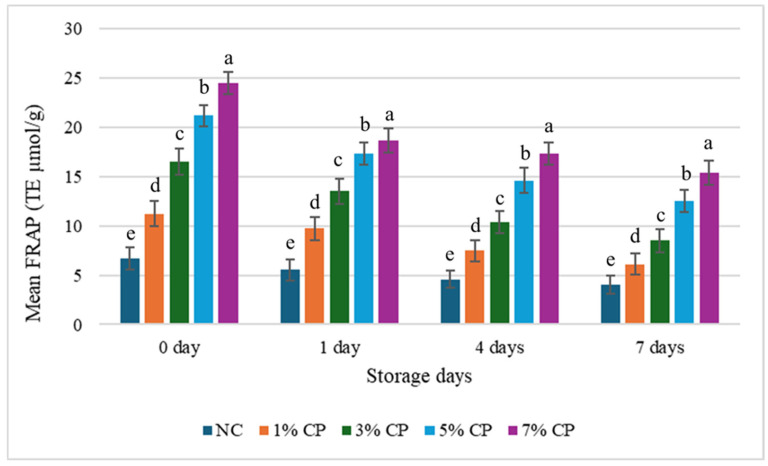
Changes in the mean FRAP value of the treatments during storage. Data are presented as mean and standard deviation as error bar. Different letters (a–e) for each day cluster represent significant differences (*p* < 0.05) among the treatments.

**Figure 3:**
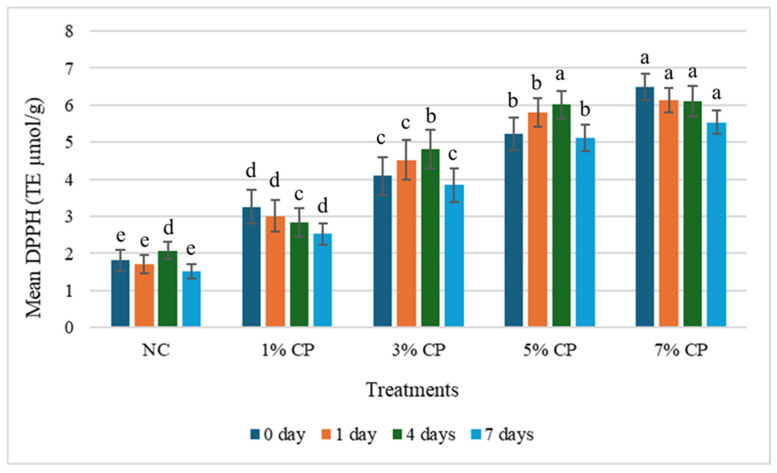
Changes in the mean DPPH value of the treatments during storage. Data is presented as mean and standard deviation as error bar. Different letters (a–e) for each treatment on each storage day represent significant differences (*p* < 0.05).

**Figure 4:**
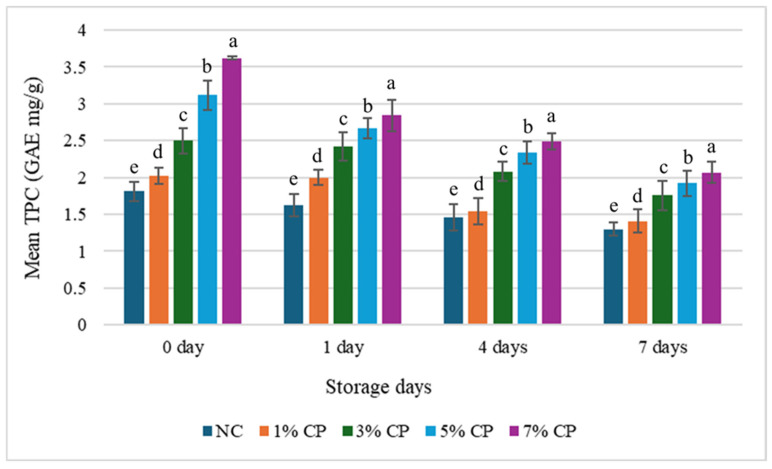
Changes in the mean TPC value of the treatments during storage. Data is presented as mean and standard deviation as error bar. Different letters (a–e) for each day cluster represent significant differences (*p* < 0.05) among the treatments.

Storage has a substantial impact on antioxidant activity and total phenolic content. All the samples were analyzed for antioxidant activity after 0, 1, 3, and 7 days of storage, and the results are presented in Figure 2, Figure 3 and Figure 4. Overall, a decrease in FRAP was observed during the 7-day storage period, with a significant difference (*p* < 0.05) between 0 and 7 days. NC samples consistently showed the lowest values, whereas 7% CP samples consistently showed the highest capacity during the 7-day storage period.

For DPPH radical scavenging activity, the values differed among treatments during storage. For NC, 1%, 3% and 5% CP, no significant changes were observed between each different storage period (*p* < 0.05). Only the 7% CP treatment showed significant differences (*p* < 0.05) in DPPH activity between day 0 and day 7. This could be attributed to the higher initial antioxidant content in the 7% CP samples, which might allow it to degrade slowly.

The TPC values showed varying trends across treatments during storage. No significant changes happened in TPC value of the NC group (*p* < 0.05). Samples containing 1% CP showed a similar TPC value at 0 and 1 day. Later, a significant drop occurred on day 4, which remained constant for the 7 days (*p* < 0.05). In the 3% CP treatment, the TPC values on days 4 and 7 were similar but significantly different from those on days 0 and 1, which also differed significantly from each other (*p* < 0.05). In the 5% and 7% CP treatments, TPC values differed significantly throughout the storage period, indicating a continuous decline in phenolic content over time (*p* < 0.05). Although the antimicrobial effect of CP was generally minimal, a ~0.8 log reduction was observed in the 7% CP sample compared to NC on day 4, suggesting a mild inhibitory effect that could be enhanced in combination with refrigeration to help control microbial growth during storage.

The decrease in antioxidant activity during storage is likely due to continuous consumption of antioxidants to combat oxidation [34]. This is supported by the increase in TBARS value over time, indicating increased ROS and free radical production. Polyphenols and other bioactive compounds in CP-treated samples help neutralise these oxidants, but as they are utilised, antioxidant activity declines. This inverse relationship between TBARS and antioxidant activity highlights the role of antioxidants in delaying oxidation during storage. The findings of Ali et al. [34] align with our results, which also reported a rapid decline in antioxidant activity during storage. Hosu et al. [35] also observed antioxidant activity in Cornelian cherry up to 19 days, and significant reductions in FRAP and TPC values were observed at 19 days compared to 0 days.

### 3.4. Microbial Growth

**Figure 5:**
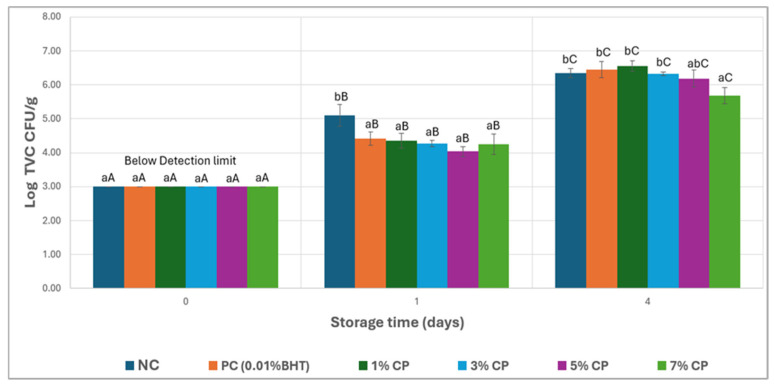
Mean TVC (log CFU/g) of the patties of different treatments during 0, 1, and 4 day storage. The error bar represent SD. Different lowercase letters (a,b) within the same storage day indicate significant differences among treatments (*p* < 0.05). Different uppercase letters (A–C) within the same treatment indicate significant differences across storage days (*p* < 0.05).

The impact of CP on microbial growth was investigated over a 4-day storage period and is presented in Figure 5. The initial microbial load, as measured by TVC, immediately after patty preparation, was uniform across all treatments at 3.00 log CFU/g. On day 1, a significant increase was observed in all treatment groups; NC had the highest TVC of 5.10 log CFU/g, whereas CP-treated samples showed slightly lower increases, with 7% CP recording the lowest at 4.26 log CFU/g. Although the results showed a potential antimicrobial effect of CP, there were no significant differences among the CP-treated groups. By day 4, further increases in microbial counts were observed, with the NC samples reaching 6.34 log CFU/g and the 7% CP sample recording the lowest count at 5.69 log CFU/g. On day 4, almost similar microbial counts were observed, except for the 5% and 7% CP treatments, which indicate a minimal antimicrobial effect of CP in products stored at 22 °C. The limited antimicrobial effect observed at 22 °C may result from both thermal degradation of the active phenolic compounds during processing and the inherently low efficacy of cherry phenolics against thermally resistant spoilage microorganisms, as reported in studies on plant-derived antioxidants in cooked meat products [16].

Previous studies reported the effectiveness of cherry powder and other natural antioxidants, such as cranberry and pomegranate, in slowing down the rate of microbial growth in raw meat samples, but mostly under refrigerated storage conditions [17,36,37,38]. High storage temperatures accelerate the metabolic activities of microorganisms, leading to rapid proliferation, and might lower the efficiency of CP’s antimicrobial activity, along with the effect of thermal processing, which can change the polyphenols’ structure and affect their functions [39,40].

### 3.5. Changes in pH

**Figure 6:**
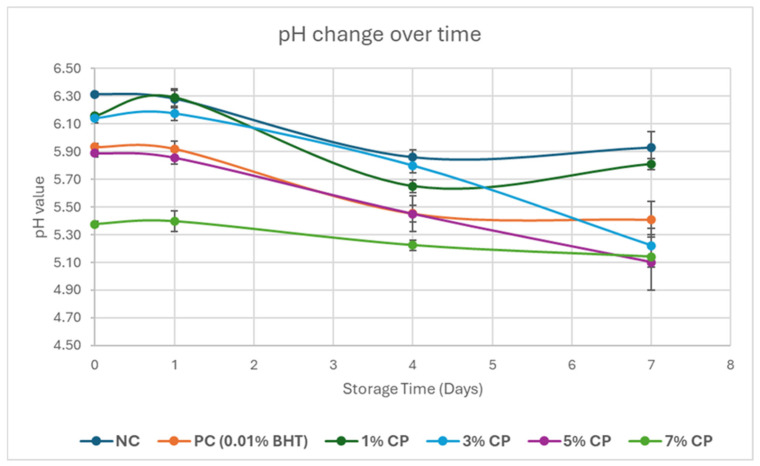
Mean pH value of the treatments during the storage period of 7 days. Error bars represent the standard deviation.

CP addition significantly affected pH over the storage period, as shown in Figure 6. On day 0, pH followed the order: NC > 1% CP ≥ 3% CP > PC ≥ 5% CP > 7% CP, ranging from 6.31 (NC) to 5.38 (7% CP). The lower pH in CP-treated samples was due to the acidic phenolic compounds present in CP [41]. Similar findings were reported by Martín-Mateos et al. [36], who found that 10% CP reduced beef pH to 5.9. By day 1, minor fluctuations were not significant (*p* > 0.05), indicating stability. By day 4, pH significantly declined (*p* < 0.05), with NC remaining highest (5.86) and 7% CP lowest (5.22). By day 7, NC, PC, and 7% CP remained stable, while 1%, 3%, and 5% CP continued decreasing, likely due to microbial activity and glycogen breakdown. This aligns with studies linking microbial growth to pH reduction in meat. Hwang et al. [42] reported a drop in beef pH from 5.52 to 5.04 over 21 days due to lactic acid bacteria. CP’s acidity initially lowered pH, which further declined due to microbial activity and organic acid production.

### 3.6. Effect of Thermal Processing on Colour

Colour (L*, a*, b*) was measured using a colorimeter to assess the impact of CP addition and thermal processing, and the results are presented in Table 2. Thermal processing significantly increased L* values (*p* < 0.05) across treatments, except for PC, likely due to protein denaturation and moisture loss [43].

Raw NC samples were the lightest, a trend that was maintained post-processing, except that PC showed the highest L*. Redness (a*) significantly decreased (*p* < 0.05) post-processing in all treatments due to myoglobin denaturation and Maillard reactions [44]. Despite CP containing anthocyanins, 5% and 7% CP samples had lower a* values (17.53, 17.59) than NC (24.92), this is attributed to polyphenol-induced metmyoglobin formation [45]. Post-processing, all samples appeared brown, with CP treatments differing significantly from NC and PC. No significant changes (*p* > 0.05) in yellowness (b*) were observed post-processing, except for NC, where b* decreased. Despite b* decreasing, NC still had the highest b* value, while 3%, 5%, and 7% CP showed the lowest values. The antioxidative properties of CP may have influenced pigment stability. These findings align with those of Kim et al. [44], who reported L* increases and a* reductions in sous vide meat, with minimal effects on b*.

### 3.7. Changes in Colour Parameters During Storage

The impact of CP treatments on colour (L*, a*, b*) changes over a 7-day storage period was observed and analyzed using one-way ANOVA, which is presented in Table 3. NC and PC showed no significant changes in L*, while 1%, 3%, and 5% CP exhibited a slight increase by day 7. No significant changes were observed in 7% CP, suggesting a threshold effect. Redness (a*) and yellowness (b*) remained stable across treatments from day 0 to day 1. By day 7, NC and PC showed no change in a*, whereas the 1% and 3% CP treatments increased slightly. No significant differences in b* values were observed across treatments.

Overall, CP improved the stability of raw meat colour but did not enhance cooked meat colour. Results align partially with prior studies [17,28,36] though L* trends differed, possibly due to variations in CP composition, storage conditions, or measurement techniques. Colour stability correlated with lipid oxidation results, as no significant changes in MDA levels were observed between day 0 and day 1. This supports the findings of Faustman et al. [45] and Zakrys et al. [46], who reported an interdependence between lipid and myoglobin oxidation, which influences pigment stability.

## 4. Conclusions

This study investigated the application of cherry powder as a natural antioxidant in meat products. It demonstrated that CP effectively enhances the oxidative stability of beef patties by reducing lipid oxidation, though its efficacy diminishes over time, particularly at higher storage temperatures. The antioxidant activity of CP declined after thermal processing but remained relatively stable during storage, supporting its potential as a functional ingredient in meat products. While CP effectively enhanced oxidative stability over the 7-day storage period, longer-term studies are needed to confirm its efficacy over typical shelf-life durations and under commercial storage temperatures. However, CP did not exhibit significant antimicrobial effects, as microbial growth was primarily influenced by storage temperature. Additionally, CP influenced meat colour, with some treatments increasing lightness and redness, suggesting potential benefits for visual appeal. In conclusion, CP is a promising natural antioxidant for improving oxidative stability and colour retention in meat products. However, optimising its application is essential to fully harness its benefits and ensure both functional and sensory quality in commercial meat formulations.

## Figures and Tables

**Table 1:** Effect of thermal processing on antioxidant activity.

	FRAP (TE µmol/g)		DPPH (TE µmol/g)		TPC (GAE mg/g)	
	Before	After	Before	After	Before	After
NC	7.55 ± 0.60	6.13 ± 0.56	3.00 ± 0.13 ^a^	1.31 ± 0.27 ^b^	2.72 ± 0.03 ^a^	1.83 ± 0.08 ^b^
1% CP	15.91 ± 0.79 ^a^	11.61 ± 0.93 ^b^	6.79 ± 0.77 ^a^	3.37 ± 0.19 ^b^	3.35 ± 0.05 ^a^	2.04 ± 0.04 ^b^
3% CP	19.30 ± 0.47 ^a^	16.13 ± 0.52 ^b^	12.16 ± 0.29 ^a^	4.18 ± 0.16 ^b^	3.66 ± 0.06 ^a^	2.43 ± 0.02 ^b^
5% CP	23.28 ± 0.13 ^a^	20.95 ± 0.27 ^b^	16.46 ± 0.19 ^a^	5.52 ± 0.32 ^b^	3.84 ± 0.03 ^a^	3.02 ± 0.10 ^b^
7% CP	26.00 ± 0.28 ^a^	24.41 ± 0.45 ^b^	20.60 ± 0.25 ^a^	6.64 ± 0.03 ^b^	4.14 ± 0.07 ^a^	3.55 ± 0.05 ^b^

^a,b^ Means with a different letter within rows are significantly different (*p* < 0.05). Data are presented as mean ± SD.

**Table 2:** Effect of thermal processing on colour parameters.

	L*		a*		b*	
	Before	After	Before	After	Before	After
NC	44.5 ± 2.13 ^b^	50.5 ± 1.91 ^a^	24.92 ± 1.70 ^a^	9.10 ± 0.56 ^b^	15.31 ± 0.74	14.07 ± 0.56
PC	48.12 ± 1.63	48.5 ± 1.58	24.12 ± 1.31 ^a^	8.52 ± 0.67 ^b^	14.52 ± 0.87	13.83 ± 0.68
1% CP	41.03 ± 2.06 ^b^	49.3 ± 1.85 ^a^	22.32 ± 1.27 ^a^	8.17 ± 0.78 ^b^	13.26 ± 0.62	12.82 ± 0.58
3% CP	42.44 ± 1.50 ^b^	48.5 ± 1.87 ^a^	19.17 ± 1.41 ^a^	7.55 ± 0.82 ^b^	11.03 ± 1.10	11.51 ± 0.54
5% CP	39.5 ± 1.72 ^b^	47.08 ± 1.80 ^a^	17.53 ± 0.86 ^a^	7.02 ± 0.59 ^b^	9.52 ± 0.73	10.81 ± 0.52
7% CP	40.51 ± 1.69 ^b^	47.5 ± 1.62 ^a^	17.59 ± 0.84 ^a^	7.12 ± 0.58 ^b^	10.10 ± 0.52	10.03 ± 0.56

^a,b^ Means with a different letter within rows are significantly different (*p* < 0.05). Data is presented as mean ± SD.

**Table 3:** Colour measurement of the patties during storage.

Colour Parameter	Treatment	Storage Time			
		Day (0)	Day (1)	Day (4)	Day (7)
L*	NC	50.95 ± 0.4 ^cA^	50.33 ± 0.5 ^abA^	50.81 ± 1.0 ^bcA^	51.77 ± 1.3 ^bA^
	PC	47.17 ± 1.3 ^abA^	51.91 ± 3.1 ^bA^	48.96 ± 1.5 ^abA^	47.96 ± 1.3 ^aA^
	1% CP	48.85 ± 0.9 ^bcA^	47.97 ± 2.5 ^abA^	54.81 ± 0.3 ^cB^	55.93 ± 0.3 ^cB^
	3% CP	47.22 ± 0.5 ^abA^	47.83 ± 0.5 ^abA^	51.21 ± 2.8 ^bcAB^	53.20 ± 0.9 ^bcB^
	5% CP	45.55 ± 0.6 ^aA^	46.51 ± 0.5 ^aA^	49.28 ± 2.1 ^abAB^	51.88 ± 2.3 ^bB^
	7% CP	45.34 ± 1.5 ^aA^	47.34 ± 1.3 ^abA^	45.61 ± 0.7 ^aA^	46.53 ± 0.8 ^aA^
a*	NC	8.11 ± 0.5 ^bcA^	8.99 ± 0.3 ^bA^	9.55 ± 0.8 ^cA^	9.75 ± 1.5 ^bA^
	PC	8.32 ± 0.3 ^cA^	8.65 ± 0.8 ^bA^	8.25 ± 0.2 ^bcA^	8.04 ± 0.2 ^abA^
	1% CP	7.42 ± 0.3 ^abA^	6.95 ± 0.9 ^aA^	8.25 ± 0.7 ^bcAB^	9.54 ± 0.6 ^bB^
	3% CP	7.40 ± 0.2 ^abAB^	6.76 ± 0.1 ^aA^	7.78 ± 0.3 ^abB^	8.80 ± 0.4 ^abC^
	5% CP	6.82 ± 0.1 ^aA^	6.88 ± 0.3 ^aA^	7.05 ± 0.5 ^abA^	8.38 ± 1.0 ^abA^
	7% CP	6.64 ± 0.2 ^aB^	6.50 ± 0.3 ^aA^	6.53 ± 0.3 ^aAB^	5.96 ± 0 ^aA^
b*	NC	13.53 ± 0.3 ^dA^	13.00 ± 0.4 ^bA^	13.55 ± 0.9 ^aA^	13.29 ± 1.0 ^bA^
	PC	13.70 ± 0.8 ^dA^	12.45 ± 0.3 ^bA^	13.14 ± 0.7 ^aA^	13.31 ± 0.5 ^bA^
	1% CP	12.64 ± 0.2 ^cdAB^	11.22 ± 1.4 ^abB^	10.51 ± 0.5 ^aA^	10.63 ± 0.2 ^aAB^
	3% CP	11.63 ± 0.2 ^bcA^	11.25 ± 0.2 ^abA^	11.11 ± 0.9 ^aA^	10.49 ± 0.6 ^aA^
	5% CP	10.66 ± 0.6 ^abA^	11.27 ± 0.5 ^abA^	10.76 ± 0.9 ^bA^	10.94 ± 1.8 ^abA^
	7% CP	9.83 ± 0.2 ^aA^	9.83 ± 0.3 ^aA^	10.66 ± 0.1 ^bAB^	10.30 ± 0.2 ^aB^

^a–d^ Means with a different letter within columns are significantly different (*p* < 0.05). ^A–C^ Means with a different letter within rows are significantly different (*p* < 0.05). Data is presented as mean ± SD.

## Data Availability

The original contributions presented in this study are included in the article. Further inquiries can be directed to the corresponding author.
